# Age-dependent effects of gut microbiota metabolites on brain resident macrophages

**DOI:** 10.3389/fncel.2022.944526

**Published:** 2022-08-22

**Authors:** Dilara Hasavci, Thomas Blank

**Affiliations:** Faculty of Medicine, Institute of Neuropathology, University of Freiburg, Freiburg, Germany

**Keywords:** microglia, macrophages, metabolites, senescence, bacteria, brain, gut microbiota, aging

## Abstract

In recent years, development of age-related diseases, such as Alzheimer's and Parkinson's disease, as well as other brain disorders, including anxiety, depression, and schizophrenia have been shown to be associated with changes in the gut microbiome. Several factors can induce an alteration in the bacterial composition of the host‘s gastrointestinal tract. Besides dietary changes and frequent use of antibiotics, the microbiome is also profoundly affected by aging. Levels of microbiota-derived metabolites are elevated in older individuals with age-associated diseases and cognitive defects compared to younger, healthy age groups. The identified metabolites with higher concentration in aged hosts, which include choline and trimethylamine, are known risk factors for age-related diseases. While the underlying mechanisms and pathways remain elusive for the most part, it has been shown, that these metabolites are able to trigger the innate immunity in the central nervous system by influencing development and activation status of brain-resident macrophages. The macrophages residing in the brain comprise parenchymal microglia and non-parenchymal macrophages located in the perivascular spaces, meninges, and the choroid plexus. In this review, we highlight the impact of age on the composition of the microbiome and microbiota-derived metabolites and their influence on age-associated diseases caused by dysfunctional brain-resident macrophages.

## Introduction

The loss of homeostasis, reduced function, and susceptibility to mortality are all signs of aging. Inflammatory, metabolic, and degenerative illnesses associated with frailty and cognitive decline are examples of age-related diseases. The molecular and cellular markers of aging in mammals have been established at a fundamental level (López-Otín et al., 2013), but they are associated with shifts in the microbiome, impacting the pace of age-related decline. The intestinal barrier, which is made up of epithelial cells, mucus, commensal bacteria, immune cells, and antibodies keeps the gut microbiota contained inside the gastrointestinal lumen in homeostatic conditions (Vancamelbeke and Vermeire, 2017). The gut microbiota releases a great diversity of metabolites to overcome this constraint and to impose effects on the host, including the central nervous system (CNS). Parenchymal microglia and perivascular, meningeal, and choroid plexus macrophages, representing non-parenchymal CNS-associated macrophages (CAMs), are among the innate immune cells of the brain (Kierdorf et al., 2019). Together, they significantly influence cerebral inflammation and can be targeted by gut-derived metabolites, especially with increasing age (Mossad and Blank, 2021). Activities connected with macrophages' highly developed lysosomal compartment are among their main tasks. Microglia and macrophages express a number of receptor families that help them degrade old, necrotic tissues and harmful substances from the circulation and their surrounding milieu (Prinz et al., 2017). The CNS is usually only mildly affected by transient activation of brain macrophages. Aging, on the other hand, is associated with chronic systemic inflammation and persistent brain macrophage activation, which can cause major physiological, behavioral and cognitive dysfunctions (Li and Barres, 2018; Mrdjen et al., 2018). In light of this, systematic identification of intestinal microbiota metabolites that reach the brain and exploration of their functions during the aging process has become a critical scientific field of interest.

## Parenchymal microglia in aging

With an estimated number of 3.5 million in the CNS, microglia account for ~10% of cells in the adult mouse brain (Lawson et al., 1990). They derive from primitive myeloid progenitors arising from the yolk sac and maintain their population in the adult brain by self-renewal (Ajami et al., 2007; Ginhoux et al., 2010; Gomez Perdiguero et al., 2013; Ginhoux and Guilliams, 2016). They have essential functions in the brain ranging from development and homeostasis to several pathologies in the CNS. Microglia regulate survival, as well as apoptosis of neuronal cells, synaptic pruning, synaptogenesis, and myelination (Li and Barres, 2018; Prinz et al., 2019) by rapidly responding to neuronal injury and invading pathogens. Therefore, microglia are considered to be a contributing factor to several disorders of the CNS during neurodevelopment and neurodegeneration (Prinz et al., 2019; Sierra et al., 2019). Under physiological conditions, microglia are highly ramified cells with long, thin processes and a small soma. Continuous motion, protrusion, and retraction of the processes allow microglia to scavenge large areas of their microenvironment for damage-associated and pathogen-associated molecular patterns (DAMPs, PAMPs). Additionally, their processes are important for the establishment of cell-cell interactions between microglia and cells of the vascular system, astrocytes, and neurons (Tam and Ma, 2014; Colonna and Butovsky, 2017). Upon infection or trauma and in neurodegenerative pathologies, microglia encounter DAMPs or PAMPs, which induce morphological conversion into an amoeboid structure allowing migration and phagocytosis at the site of injury (Nimmerjahn et al., 2005; Tremblay et al., 2011). Besides morphological changes, an alteration of gene expression was observed, resulting in increased expression of major histocompatibility complex II (MHCII) antigens. Further, activated microglia release pro-inflammatory cytokines amplifying the inflammatory response (Hayes et al., 1987; Kim and Joh, 2006). While released pro-inflammatory cytokines, including members of the interleukin (IL) family, like IL-1β and IL-6 or tumor necrosis factor-α (TNF-α), are intended to prevent further damage to cells of the CNS, elevated levels of these cytokines can also damage glial cells and neurons. Therefore, chronically activated microglia or an imbalance in release of pro- and anti-inflammatory cytokines is considered a contributing factor to development and progression of neurodegenerative diseases (Smith et al., 2012). While the role of microglia in neurodegenerative disease and during aging of the brain is undisputed, the functional dynamics of microglia during aging remain elusive. Microglia in aged, healthy individuals display dystrophic morphologies showing fragmentation and retraction of processes, less branching, and de-ramification, all associated with senescence (Sheng et al., 1998; Miller and Streit, 2007). The activation of microglia in an age-dependent manner was already described three decades ago in aged non-human primates (Sheffield and Berman, 1998), rodents (Perry et al., 1993; Ogura et al., 1994), and humans (Streit and Sparks, 1997). In addition, a downregulation of sensome genes was observed, resulting in reduced ability to survey the brain parenchyma (Streit et al., 2004; Hickman et al., 2013). Additionally, an increase of microglial MHCII expression has been observed in aged non-human primates, suggesting a higher sensitivity to different stimuli (Sheffield and Berman, 1998). Recent studies also revealed that microglia of the aged brain release higher baseline levels of pro-inflammatory cytokines, such as TNFα, IL-1β, IL-6, and IL-12b, as well as anti-inflammatory mediators including TGFβ1 and IL-10 counterbalancing each other to maintain a steady state. The same is observed in brains from humans with Alzheimer's disease (AD) (Streit et al., 2004; Miller and Streit, 2007; Costa et al., 2021).

## Non-parenchymal macrophages in aging

Besides microglia, non-parenchymal macrophages occupy different strategic niches in the choroid plexus (cpMΦ), subarachnoid space and pia mater (mMΦ), and perivascular (pvMΦ) spaces, thereby covering the whole CNS. They can be differentiated by their localization and expression of overlapping, as well as unique marker genes (Prinz et al., 2017). Fate-mapping studies regarding the ontogeny of non-parenchymal macrophages reveal that pvMΦ, as well as mMΦ mainly derive from embryonic hematopoietic precursors and constantly self-renew (Goldmann et al., 2016). CpMΦ however, originate from both adult hematopoietic stem cells (HSCs) and embryonic myeloid progenitors (Goldmann et al., 2016; Prinz et al., 2017). Brain-resident macrophages not only differ in localization from microglia, but also in their transcriptomic profile. CpMΦ, mMΦ, and pvMΦ, but not microglia, highly express mannose receptor (CD206), which is involved in recognition of pathogens and endocytosis (Linehan et al., 2000; Galea et al., 2005; Faraco et al., 2016). Further, non-parenchymal macrophages in the brain are CD163 positive and express CD45 at a higher level than microglia (Chinnery et al., 2010; Goldmann et al., 2016). Besides transcriptional distinction from microglia, single-cell RNA-sequencing shows that the gene expression differs between populations itself. PvMΦ and mMΦ both can be distinguished from cpMΦ by expression of lymphatic vessel endothelial hyaluronic receptor-1 (LYVE1) (Zeisel et al., 2015; Goldmann et al., 2016). In the healthy CNS, brain-resident macrophages react to DAMPs and PAMPs *via* receptors on their surface by induction of pro-inflammatory cascades. Similar to microglia, the signaling cascade drives morphological changes toward an amoeboid phenotype and leads to elevated levels of pro-inflammatory cytokine secretion (Prinz and Priller, 2014). The different subsets are suggested to be involved in monitoring of the CNS and clearance of cellular debris and pathogens (Nayak et al., 2012; Kierdorf et al., 2019). During aging, senescent macrophages progressively lose their ability of a pro-inflammatory response as well as phagocytic and chemotactic functions (Shaw et al., 2013).

## Influence of the microbiome on CNS macrophages

Recent research has found that the microbiome influences the properties and function of CNS macrophages. Studies in germ-free (GF) mice revealed the importance of the microbiome in microglial development and maturation, as well as function in the adult brain. Microglia from adult GF and specific pathogen-free (SPF) mice display different morphologies including branch points, dendrite length, segment number, and cell volume. Additionally, the transcriptomic profile of microglia in GF mice shows a downregulation of several genes involved in cell activation and induction of immune response (Erny et al., 2015). The lack of mature gene expression in these microglia is linked to the absence of microbiota in the gut intestinal tract and disrupts their ability to respond to immunostimulants (Erny et al., 2015). When challenged with lipopolysaccharides (LPS), microglia from GF mice show a decreased expression of IL-1β, IL-6, and TNF-α and reduced amoeboid morphology, associated with activation. These findings suggest a crucial role of the microbiota in microglial maturation and function during immune response (Erny et al., 2015). It is interesting to note that microglia also have sex- and age-dependent responses to microbiota. For example, male mice's microglia are more sensitive to microbiome loss in the embryonic stage, but female mice's lack of microbiota causes the most significant changes in transcriptomic profiles during maturation. Dimorphic alterations in microglial markers reveal a link between gut microbiota and gender-biased CNS diseases (Thion et al., 2018). In fact, several neurological diseases associated with microglial dysfunction are accompanied by dysbiosis, an imbalance of the microbial community in the gastrointestinal tract (GIT) (Hsiao et al., 2014; Sampson et al., 2016). One of the most prominent neurological diseases associated with aging is AD (Nichols et al., 2019). Men and women with AD have different cognitive and psychiatric symptoms, and women experience faster cognitive deterioration after being diagnosed with AD dementia (Ferretti et al., 2018). In AD mice, fecal microbiomes show striking increases in *Verrucomicrobia* and *Proteobacteria* as well as significant decreases in *Ruminococcus* and *Butyricicoccus*. This indicates altered microbiota composition and diversity, while the observed reduced SCFA levels point toward alterations in several metabolic pathways (Zhang et al., 2017). Similar to the findings in AD mouse models, microbial taxa are also altered in AD patients compared to control subjects. However, the data seem somewhat conflicting because the phylum *Bacteroidetes* was reported to be either increased (Vogt et al., 2017) or slightly decreased in AD patients (Zhuang et al., 2018), depending on the study. One of the key symptoms of AD, the amyloid-beta (Aβ) burden, is influenced by the gut microbiome. Studies in GF and SPF 5x familial AD (5xFAD) mice (Oakley et al., 2006), a mouse model for AD, uncovered a reduced pathology in GF (Mezö et al., 2020) or antibiotics-treated animals (Guilherme et al., 2021) marked by lower Aβ plaque burden. In support of these findings, deficiency of bacteria in the GIT leads to an elevated uptake of Aβ by microglia (Mezö et al., 2020). These results could only be observed in young mice and seem to be age-dependent since the Aβ load in aged mice becomes indistinguishable between GF and SPF mice. Biological age and gender seem to be important factors influencing microglial function under pathological and non-pathological conditions. Similar studies have focused on non-parenchymal macrophages in the CNS. The function of CNS-associated macrophages (CAMs) was analyzed in 5xFAD mice under SPF and GF housing conditions. It was revealed that cpMΦ are responsive to the absence of the microbiome. Additionally, gut microbiota influences the Aβ uptake of pvMΦ in 5xFAD mice (Sankowski et al., 2021). Further, CAMs in GF mice lack an appropriate response to immunostimulants typically characterized by expansion and expression of CD74. These findings suggest that the innate immune response depends on the presence of microbiota in the GIT and absence of the microbiome disrupts innate immunity (Sankowski et al., 2021). The gut microbiota has a major influence on CNS macrophages during development until adulthood and their metabolites govern the inflammatory response in the CNS, which is mediated by macrophages. Brain macrophages may act as crucial mediators between the gut microbiome and CNS disorders as seen in the case of AD.

## Gut-brain-axis in aging

Countless bacteria, viruses, yeasts, bacteriophages, and fungi inhabit our bodies. While microorganisms can be found on almost all environmentally exposed surfaces of our body, the gastrointestinal tract (GIT) shows the highest number and density of microbiota. These communities have significant impact on numerous physiological mechanisms, such as function of the immune system and metabolism (Zhuang et al., 2018; Dabke et al., 2019). The gut modulates several functions in the brain by bacteria-derived metabolites, hormones, and neuroactive substances reaching the CNS *via* the vagus nerve, enteric nervous- and circulatory system, and immune system (Long-Smith et al., 2020). Conversely, the central nervous system modulates gut function through the hypothalamic-pituitary-adrenal axis, as well as the autonomic nervous system (Mart'yanov et al., 2021). The composition and activity of microbiota in the GI tract is highly dynamic and influenced by internal factors, such as age and genetics of the host, as well as external factors including dietary changes, and over-use of antibiotics (David et al., 2014; de la Cuesta-Zuluaga et al., 2019; Vich Vila et al., 2020). Accordingly, each microbiome is unique and remarkably varies in composition between individuals, even under healthy conditions. An alteration of the bacterial community in the gut, medically termed dysbiosis, is strongly associated with several host diseases including disorders of the CNS (Sudo et al., 2004; Bäckhed et al., 2005; Clarke et al., 2013; Sommer and Bäckhed, 2013; Hsiao et al., 2014; Singh et al., 2016; Fang et al., 2020). The blood-brain barrier (BBB), which connects the CNS to the periphery, is crucial for the protection against pathogens and potentially neuron-harming immune reactions (Daneman and Prat, 2015). This function is achieved by precise regulation of in- and efflux of molecules, ions, and cells (Engelhardt and Liebner, 2014). Bacteria-derived metabolites are also able to pass the BBB and are important in CNS development and homeostasis, but also play a role in the development and progression of diseases of the CNS (Heijtz et al., 2011; Rutsch et al., 2020; Mei-Sin Tran and Mohajeri, 2021). The bidirectional communication between the CNS and the intestinal microbiome is referred to as the gut-brain axis (Sudo et al., 2004; Skonieczna-zydecka et al., 2018) and is indispensable for maintenance of homeostasis of the gut and CNS (Martin et al., 2018; Cryan et al., 2019). There is growing evidence, that the BBB is significantly affected by the composition of gut bacteria. Germ-free mice, as well as mice treated with broad-spectrum antibiotics, display dysregulated tight-junctions resulting in enhanced BBB permeability (Braniste et al., 2014). Importantly, during the aging process and even more so in the context of neurodegenerative disorders, the BBB is getting increasingly more defective, which enables the entry of neurotoxic gut-derived products into the brain where they elicit inflammatory and immune responses (Zlokovic, 2011). According to reports, with increasing age, the brain endothelium gradually becomes dysfunctional, linked to abnormal BBB alterations (Cai et al., 2017; Edwards et al., 2019). The extracellular matrix (ECM) of the basal membrane or basal lamina, which is thought to be homogeneous and thin, covers the brain endothelium. In the course of aging in healthy humans, laminin concentrations fall while collagen IV concentrations raise, increasing the ECM's thickness (Candiello et al., 2010). ECM promotes the production of occludin, a member of tight junction proteins, which helps to maintain BBB integrity. Thus, alterations in the ECM lead to disruptions in the BBB, causing increased permeability (Candiello et al., 2010; Sanchez-Covarrubias et al., 2014). As we age, additional physiological functions start to deteriorate, which becomes evident in reduced intestinal function, as well as declining immunity, and significant changes in the microbial composition in the GIT. Many of these age-related phenomena can be traced back to dysbiosis. The microbiome of aged individuals is characterized by decreased diversity, together with an increased abundance of bacteria associated with pro-inflammatory effects microorganisms, comprising including *Enterobacteriaceae* and *Clostridia* and reduced numbers of *Bifidobacterium* and *Lactobacillus*, both beneficial genera (O'Toole and Jeffery, 2015; Odamaki et al., 2016). Recent studies suggest an association between age-related changes of the gut-microbiome and several pathologies and diseases, e.g., cancer, insulin resistance, diabetes, cardiovascular disease, and neurodegenerative diseases (Gérard and Vidal, 2019; Vivarelli et al., 2019; Cheng et al., 2020; Fang et al., 2020; Kazemian et al., 2020).

## Gut metabolites in aging

Bacteria in the GIT contribute greatly to the degradation of indigestible compounds by synthesis of a variety of enzymes (Rowland et al., 2018). Besides support of digestion, gut microbiota produce numerous metabolites. These bioactive substances have profound effects on the host including the regulation of metabolic pathways by, for example, feedback mechanisms, absorption of nutrients and the microbiota composition itself (Perino et al., 2021; de Vos et al., 2022). Advanced metabolomics tools uncovered a large number of bacteria-derived metabolites including short-chain fatty acids (SCFAs), choline metabolites and bile acids (Tang et al., 2019). As the natural consequence of age-associated changes in the composition of microbiota in the gut, levels of bacteria-derived metabolites are altered. Metabolomics of fecal matter derived from aged individuals with age-matched or mismatched gut-microbiome revealed significant differences in eight metabolites (Yoshimoto et al., 2021). Three of these metabolites were enriched in stool samples from individuals with an elderly gut-type. Trimethylamine (TMA) and its precursor choline, as well as propionic acid were all abundantly present. Choline and TMA also induced the expression of interleukin (IL) 8 and IL-21, both pro-inflammatory cytokines inducing colorectal cancer cell growth and survival (Rubie et al., 2007; Mager et al., 2016). *In*-*vitro* analyses of these metabolites revealed that they negatively influence the mucosal layer of the gut epithelial layer by suppression of tight junction-related genes in human normal colonic epithelial cells (HCoEpiCs). Further, cholic acid, enriched in the younger microbiome group, leads to an upregulation of these genes in HCoEpiCs (Yoshimoto et al., 2021). Consequently, with increasing age, various metabolites, either from dietary sources or generated directly by the gut microbiota, are able to reach the blood circulation and ultimately the brain. This process is inhibited at younger ages when the integrity of the gut epithelium, as well as the BBB are still intact. We have verified N6-carboxymethyllysine (CML) as one of these metabolites, which is found in processed food and shows higher levels in human sera and brains with increased age. The greater levels of CML in old mice were mediated by a microbiota-dependent rise in intestinal permeability and triggered microglial reactive oxygen species (ROS) production, which inhibited mitochondrial function and impaired ATP production and storage (Mossad et al., 2022). While these data suggest that elevated levels of certain age-associated metabolites contribute to increased gut permeability during aging *in-vitro*, human *in-vivo* studies are conflicting. More recently, *in-vivo*, as well as *ex-vivo* human studies showed no significant differences in permeability of the small intestine, colon, or whole gut between young and aged individuals (Wilms et al., 2020). On the contrary, Man et al. discovered a correlation between aging and changes in permeability of the small intestine (Man et al., 2015). Further assessing the significance of aging with respect to intestinal barrier function will be needed to form a more comprehensive view on gut permeability in aged individuals.

## Short-chain fatty acids

SCFAs are saturated fatty acids and products of anaerobic bacterial fermentation of dietary fibers (Louis and Flint, 2009). They mainly comprise of acetate, propionate and butyrate (Fernandes et al., 2014; Luu et al., 2019). Generally, the role of SCFAs in the physiology of the host involves maintenance of gut barrier integrity and support of GIT homeostasis (Donohoe et al., 2011; Den Besten et al., 2013). Most of the SCFAs in the gut are absorbed by colonocytes, where they influence colonic blood flow, water and salt uptake, and gut motility (Salminen et al., 1998). Further, SCFAs are able to reach the BBB *via* the bloodstream, where they can directly act on the integrity of the BBB (MacFabe, 2012). Studies in colonized germ-free mice showed the influence of SCFAs on the permeability of the BBB. By colonization of germ-free animals with butyrate-producing bacteria, tight-junction proteins are upregulated leading to decreased BBB permeability (Braniste et al., 2014). Fecal levels of SCFAs are significantly reduced in aged individuals, which can be attributed to age-related changes in microbial composition (Salazar et al., 2013, 2019). Surprisingly, the bacterial communities in young and elderly humans show both similar numbers of SCFA producing bacteria (Salazar et al., 2019). This divergent trend in levels of bacteria and SCFAs suggests a reduction in metabolic activity of gut-resident bacteria with age. Reduced levels of SCFAs are associated with several diseases such as AD and Parkinson's disease (Marizzoni et al., 2020; Chen et al., 2022). Like other metabolites, SCFAs can cross the BBB and interact with microglia. High levels of SCFAs in the brain inhibit the inflammatory response of peripheral monocytes, but low levels, as reported in the elderly, are related with systemic CNS inflammation (Wenzel et al., 2020). At the same time, data strongly suggest a crucial role for SCFAs in modulating the BBB integrity itself, as seen in the cases of the SCFA butyrate (Park and Sohrabji, 2016) and propionate (Hoyles et al., 2018). Augmenting the access of butyrate and propionate to the BBB during aging should antagonize the unfavorable consequences of a compromised BBB for brain function.

## Trimethylamine N-oxide

Choline-derived Trimethylamine N-oxide (TMAO) is an amine oxide and osmolyte, which is enriched in certain foods, such as marine crustaceans and fish (Velasquez et al., 2016). Besides direct uptake of TMAO through diet, the microbiota in the GIT is able to produce trimethylamine (TMA), the precursor of TMAO. These bacteria process choline, lecithin and carnitine derived from red meat and other animal products to TMA, which is converted into TMAO by host hepatic enzymes (Koeth et al., 2014). High levels of TMAO, regardless of the source, are mostly associated with cardiovascular disease (Tang et al., 2013). However, several other age-related pathologies have been reported to be closely linked to elevated TMAO levels including arteriosclerosis (Tang et al., 2013), Alzheimer's disease (Xu and Wang, 2016) and cancer (Guertin et al., 2017). Levels of circulating TMAO have been found to be significantly increased in aged humans, as well as in mice and rats (Li et al., 2017, 2018) suggesting a correlation between age and TMAO production. Studies in mice showed that treatment with TMAO negatively influences cognition and working memory (Li et al., 2018; Govindarajulu et al., 2020). Further, TMAO promotes neuronal senescence and synaptic damage while downregulating the expression of proteins associated with synaptic plasticity and inhibition of the mTOR signaling pathway (Li et al., 2018). These TMAO-induced processes contribute, at least partially, to age-related deterioration of the brain and cognitive dysfunction. Individuals with mild to severe cognitive impairment and AD patients show elevated levels of TMAO in their cerebrospinal fluid (CSF), indicating an involvement of TMAO in neurological decline (Vogt et al., 2017). This hypothesis is strengthened by a recent study showing plasma levels negatively correlating with cognitive function, which is mediated by inflammatory signaling in microglia (Brunt et al., 2021). Additionally, an increase of oxidative stress driving mitochondrial impairments has been observed in the hippocampus of TMAO-treated mice. While the negative effects of ROS are typically neutralized by antioxidants, an imbalance leads to oxidative stress, which is associated with a variety of age-related diseases (Liguori et al., 2018). In a rat model similar observations were made when treatment with TMAO raised pre-operative and post-operative plasma levels of TMAO, which exacerbated microglia-mediated neuroinflammation (Meng et al., 2019). Contrasting these detrimental effects, data using an integrated *in vitro/in vivo* approach indicate that TMAO can improve BBB integrity and shield the brain from inflammatory insults by increased expression of the tight junction regulator annexin A1. Chronic TMAO exposure would thus limit microglial responsiveness in a brain region-specific way and protect the aging brain from inflammatory insults (Hoyles et al., 2021). This assumption is based on data indicating that microglia of the entorhinal cortex are sensitive to the presence of TMAO while microglia in the neighboring hippocampus appear not to be affected (Hoyles et al., 2021).

## δ-Valerobetaine

Most recently a precursor of TMAO, δ-Valerobetaine, a small metabolite, was the subject of several studies investigating the influence of this gut-derived metabolite on the host. The production of δ-Valerobetaine in mammals relies on gut-resident bacteria (Servillo et al., 2018; Liu et al., 2021). Incubation of monocultures of gut-resident bacteria with the precursor of δ-Valerobetaine revealed that several taxa including *Lactobacilli, Escherichia coli*, and *Bifidobacterium longum* are able to convert Nε, Nε, Nε-trimethyllysine to δ-Valerobetaine (Liu et al., 2021). δ-Valerobetaine is found in the circulation and different organs including brain and liver of SPF mice, but absent in GF mice. Conventionalization of GF mice with bedding of SPF mice, however, leads to comparable levels of δ-Valerobetaine in the serum and organs (Liu et al., 2021). Mossad et al. showed the age-dependency of δ-Valerobetaine levels in serum and brain of mice and humans. The heightened levels of δ-valerobetaine in aged individuals were reversed by fecal matter transplantation (FMT) from young to aged mice, confirming the source of δ-valerobetaine as the bacteria in the GIT. Further, treatment of young mice with δ-valerobetaine revealed that high concentrations of this metabolite in the serum are strongly associated with cognitive impairment and negatively impacts learning and memory processes (Mossad et al., 2021). The role of δ-valerobetaine in age-related cognitive impairment remains elusive. In hepatic cells, however, δ-valerobetaine decreases cellular carnitine and mitochondrial acyl-coenzyme A, both crucial for mitochondrial fatty acid oxidation. This study also showed that high δ-valerobetaine concentrations correlate with levels of di- and triacylglycerides in several organs, including the brain. Both of which are associated with AD (Wood et al., 2015; Zhang et al., 2020).

## Amino acid metabolites

Amino acids are a crucial part of macronutrients in mammalian diets and essential for production of peptides and synthesis of bioactive molecules involved in signaling pathways and metabolism (Wu et al., 2014). Gut-resident bacteria influence bioavailability by utilizing amino acids derived from the host or dietary sources for synthesis of various metabolites (Wikoff et al., 2009; Dai et al., 2011). Importantly, several nutritionally essential amino acids can be synthesized *de novo* by bacteria in the GIT (Metges, 2000). Particularly interesting is tryptophan, an aromatic amino acid, which is derived from protein-rich foods (Shabbir et al., 2013). The metabolism of tryptophan by gut-resident bacteria contributes greatly to the synthesis of 5-Hydroxytrypanim (serotonin), tryptamine, and kynurenines, all involved in the bi-directional communication of the gut-brain axis (Slominski et al., 2002; Agus et al., 2018). Among the already mentioned bioactive molecules, tryptophan-metabolizing bacteria synthesize indoles, ligands for the aryl hydrocarbon receptor (AhR) important in neurological processes. AhR signaling is involved in several neurological processes. In microglia, AhR signaling indirectly controls astrocyte activation *via* regulation of transforming growth factor β (TGF-β) and vascular endothelial growth factor B (VEGF-B) expression (Rothhammer et al., 2018). Stimulation of astrocytes *via* indoles binding to the AhR receptor has been shown to reduce severity of multiple sclerosis (MS) symptoms and brain inflammation in a MS mouse model (Rothhammer et al., 2016). Indoles can cross the BBB under non-pathological conditions as seen from experiments where oral administration of indole increased adult neurogenesis in WT C57BL/6J mice *via* AhR signaling (Wei et al., 2021). This observation makes it very likely that indoles can also reach the brain during aging and modify microglia function. In support of this assumption, it has to be noted that the aging brain displays elevated type I interferon-levels, which can be sensed by microglia and favor AhR expression (Baruch et al., 2016; Deczkowska et al., 2017).

## Lipopolysaccharide (LPS)

Lipopolysaccharides are crucial elements of gram-negative bacteria's outer membrane. They are substantial amphipathic glycoconjugates, often made up of a core oligosaccharide and a distal polysaccharide linked to a hydrophobic lipid domain. Due to the fact that these molecules include both lipid and sugar molecules, they are sometimes referred to as lipoglycans (Kuhn, 2019). Dysbiosis of the gut microbiota associated with aging increases the paracellular permeability of the gut and permits LPS to escape into the bloodstream. In addition, the BBB becomes more permeable with age, allowing circulating pro-inflammatory LPS to enter the brain tissue (Thevaranjan et al., 2017). In a mouse model with accelerated aging, microglia also exhibit greater proliferation along with an elevated and uncontrolled inflammatory response to peripheral inflammatory stimuli, such as LPS (Raj et al., 2015). LPS is further a well-known ligand for toll-like receptors (TLRs) 4 that, when activated, causes microglia to generate pro-inflammatory cytokines (Papageorgiou et al., 2016). Functional transcriptomics predicted that LPS is the most important upstream regulator of lipid droplets in microglia (Marschallinger et al., 2020). A hydrophobic core of neutral lipids, primarily triglycerides and cholesteryl esters, surrounds a phospholipid monolayer adorned with proteins known to govern lipid droplet function. These lipid-droplet-accumulating microglia, present in aged brains, have phagocytosis abnormalities, create more ROS, and release higher levels of pro-inflammatory cytokines (Marschallinger et al., 2020). The decreased phagocytic ability of lipid droplet-containing microglia proposes a putative feedback loop in which excessive lipid droplet build-up in these cells slows phagocytosis rates. In the aging brain, lipid droplets are not restricted to microglia only but are also present in perivascular and meningeal macrophages. There is no current data on the functional consequences for both cell types available (Shimabukuro et al., 2016).

## Conclusions

There is now increasing evidence that metabolites produced in the gut can enter the brain and impact brain macrophages. In consequence, it is important to better understand the underlying mechanisms of age-related dysbiosis, which causes changes in gut-derived metabolites and ultimately influence the CNS, as well as immune and endocrine responses of the host. Several studies have found that microbial metabolites can affect gut–brain responses, affecting the morphology and function of brain macrophages. These changes include their polarization and phagocytic capacity, which, in turn, controls behavior and emotional processes. The notion that age-related changes in gut microbial ecology and function, as well as the participation of specific bacterial species, might be predictive for pertinent clinical problems is a basic issue that has to be resolved. A roadblock in today's microbiota-based biomedical research is the modest and long-term impact on psychological and cognitive performance. Probiotic and microbiota-based therapies may take months to years to affect neuropsychiatric illnesses, while the influence of the microbiome on host coagulation can be seen fairly rapidly. In this regard, integrating multiple metabolomic, metagenomic, metatranscriptomic, and proteomic methods to facilitate a more detailed portrayal of the multifaceted microbial ecosystem and key metabolites in order to verify their therapeutic potential as adjuvant in the treatment of age-related gut–brain axis pathologies will be a promising strategy.

## Author contributions

Both authors listed have made a substantial, direct, and intellectual contribution to the work and approved it for publication.

## Funding

We are grateful for the support by the DFG (SFB/TRR167 NeuroMac) to DH and TB.

## Conflict of interest

The authors declare that the research was conducted in the absence of any commercial or financial relationships that could be construed as a potential conflict of interest.

## Publisher's note

All claims expressed in this article are solely those of the authors and do not necessarily represent those of their affiliated organizations, or those of the publisher, the editors and the reviewers. Any product that may be evaluated in this article, or claim that may be made by its manufacturer, is not guaranteed or endorsed by the publisher.
